# Resource use and economic burden of eye injuries in Southern Finland

**DOI:** 10.1007/s00417-021-05399-3

**Published:** 2021-09-06

**Authors:** Ahmad Sahraravand, Anna-Kaisa Haavisto, Tiina Leivo

**Affiliations:** Ophthalmology, University of Helsinki, and Helsinki University Hospital, Haartmaninkatu 4 C, 00290 Helsinki, Finland

**Keywords:** Costs, Economic burden, Eye injuries, Resources, Southern Finland

## Abstract

**Objective:**

To estimate resource use and the costs of eye injuries in 2011–2012 in the Helsinki University Eye Hospital (HUEH), which covers 1.6 million people in Southern Finland.

**Methods:**

This population-based study consisted of all new patients (1,151) with eye injuries in one year. The data were from hospital records, internal HUEH accountancy, and prospectively from questionnaires. The costs of direct health care, transportation, and lost productivity were obtained and estimated for the follow-up period of three months. The estimated future costs were discussed.

**Results:**

During the follow-up, the total cost was 2,899,000 Euros (EUR) (= EUR 1,870,300/one million population), including lost productivity (EUR 1,415,000), direct health care (EUR 1,244,000), and transportation (EUR 240,000). The resources used included 6,902 days of lost productivity, 2,436 admissions and transportations, 314 minor procedures, 313 inpatient days, 248 major surgeries, and 86 radiological images. One open globe injury was the costliest (EUR 13,420/patient), but contusions had the highest overall cost (EUR 1,019,500), due to their high occurrence and number of follow-ups.

**Conclusions:**

Eye injuries cause a major burden through high costs of direct health care and lost productivity: the imminent costs were EUR 1,870,000/one million population, and the future costs were estimated to EUR 3,741,400/one million population. Prevention remains the main factor to consider for better cost-efficiency.

**Supplementary Information:**

The online version contains supplementary material available at 10.1007/s00417-021-05399-3.


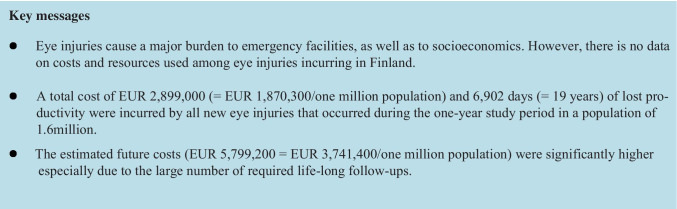


## Introduction

Eye injuries occur universally in everyday activities and are among the leading causes of monocular blindness in the world [1]. They are mostly predictable and hence, preventable; many of their risk factors have been identified which has led to favorable outcomes through eye safety recommendations [2–5]. However, they cause an extensive burden to emergency facilities, as well as to socioeconomics through reduced or permanently lost work ability. The impacts of lost productivity may be short term: for example, parents may have to miss work to take care of an injured child; or long term, for example, life-long follow-ups.

It is estimated that 55 million (= 9,500/1,000,000 population) eye injuries occur in the world each year and that 750,000 (= 130/1,000,000 population) injuries require hospitalization [1].

To evaluate the influence of eye injuries, and to set policies for priorities, it is crucial to have information on not only the prevalence and causes, but also on the costs incurred by eye injuries.

Existing reports on the costs incurred by eye injuries from different countries are mostly outdated. Moreover, the comparability of these studies is limited due to different insurance and compensation policies, or developmental diversity. Many studies have a narrow focus; for example, they report the expenses of preventable and minor [6], or serious eye injuries [7, 8], or those caused intentionally, at work, or among children [7, 9, 10].

Few studies have reported the cost of all eye injuries. Mönestam estimated a total cost of eye injuries of SEK 1,300,000 (= ca. EUR 197,400 [11]) in a hospital with a population base of 115,000 in Northern Sweden in 1986 [12].

Many studies have addressed inpatient days and lost productivity days [13–17]. Median hospitalization costs of ocular injuries in Texas were between USD (United States Dollars) 34,576 and USD 55,409 (= EUR 26,000 and EUR 42,000 [11]) for a 2- to 4-day hospital stay during 2013–2014 [16].

In Finland in 1980–1986, perforating eye injuries caused 5% of permanent disability and inability to work [8].

To our knowledge, no recent studies have described direct and indirect costs, the use of resources, or the costs incurred by serious and minor eye injuries in European countries.

The strength of our study is that it is a population-based study. The aim of this study was to estimate the economic burden and resources used for eye injuries in Southern Finland from a societal perspective, including direct health care costs, direct non-health care costs, and indirect costs.

## Materials and methods

In this population-based study, the participants consisted of all new patients with an eye injury admitted to the Helsinki University Eye Hospital (HUEH) emergency department (ED) over one year (1 May 2011 to 30 April 2012).

Data was obtained prospectively from patient questionnaires and retrospectively from hospital records. These included information on patient demographics, symptoms, detailed physical eye examinations, treatments, use of resources, and the cause of and events leading to the eye injury. The follow-up period was three months. We divided the cases into three age groups: children aged under 17 years, adults aged 17–60 years, and the elderly aged over 60 years. We classified the ICD-10-coded (International Classification of Diseases-10) cases as BETTs (Birmingham Eye Trauma Terminology system) [18, 19] and into seven diagnostic groups according to their primary diagnosis. These were chemical and burn eye injuries, contusions, orbital fractures, open globe injuries (OGI), optic nerve injuries (ONI), superficial minor eye injuries, and eyelid wounds with or without lacrimal injuries (Table 1) [20–22].

**Table 1:** Mean direct, indirect, and total cost per patient during follow-up of eye injuries by different diagnostic groups

	Patients	Direct health care cost	Direct non-health care cost	Patients	Lost productivity	Indirect cost	Mean total cost
Diagnostic group	(N)^1^	(€/pt)^2^	(€/pt)	(n)^9^	(days)	(€/pt)	(€/pt)
Chemicals^3^	*137*	670	215	*106*	982	1,900	**2,355**
Contusions	*273*	1,240	300	*208*	2,922	2,880	**3,735**
Fractures^4^	*50*	3,440	427	*40*	532	2,730	**6,050**
OGI^5^	*29*	8,300	470	*20*	658	6,740	**13,420**
ONI^6^	*4*	6,530	270	*4*	72	3,690	**10,490**
Superficials^7^	*604*	380	135	*409*	1,466	730	**1,010**
Wounds^8^	*54*	2,680	210	*38*	270	1,460	**3,915**
*All*	***1,151***	**1,080**	**210**	***825***	**6,902**	**1,715**	**2,515**

^1^* N* number of patients; ^2^*€/Pt* Euros per patient; ^3^chemical and burn injuries; ^4^orbital fractures; ^5^open globe injuries; ^6^optic nerve injuries; ^7^superficial minor injuries; ^8^eyelid wounds and/or canalicular injuries; ^9^number of patients with lost productivity

The costs were grouped into direct and indirect costs. Direct costs included health care costs and non-health care costs. Direct health care costs included outpatient visits, hospitalizations, major surgeries, minor procedures, and medication and radiology costs. Direct non-health care costs included transportation costs. Indirect costs included the costs of lost productivity.

We derived the unit cost for outpatient visits, hospitalizations, major surgeries, and minor procedures from the internal HUEH cost accounting data [23].

Outpatient admissions were calculated for the three age groups as well as for all diagnostic groups. This included number of admissions of the first visits in the Emergency Department (ED) + the first control visits in ED clinic + the following possible follow-up visits at an eye sub-specialty clinic during three months of follow-up.

The cost of hospitalizations was based on the number of inpatient days.

The major surgeries included the services of an anesthesiologist (sedation or general anesthesia). The unit costs were obtained from resourced-based internal HUEH cost accounting data. The unit cost was different for an emergent and for an elective operation (Table 2). In the case of several simultaneous operations, the cost included the most expensive procedure added to half of the sum of the other procedures.

**Table 2:** Unit and total costs during follow-up of eye injuries by age group in Southern Finland over period of 1 year. Population base 1.6 million, number of injuries 1151, follow-up 3 months

Resource	Patients	Age groups	Cost component	Cost component units	Unit cost	Total cost	Total cost/1 M
	(*n*)		(*n*)		(€/unit)	Thousands (€)	Thousands (€)
**Indirect costs (lost productivity)**	**825**		**6902**	**Days**	**205**	**1,415**	**913**
	95	Children	805			165	
	702	Adults	5,629			1,154	
	28	Seniors	468			96	
**Direct health care costs**						**1,244**	**802**
**Outpatient visits**	**1151**		**2,436**	**Visits**	**150–250**	**503**	**325**
	202	Children	444			91	
	831	Adults	1,722			357	
	118	Seniors	270			55	
**Major operations**	**149**		**248**	**Operations**	**495–9,525**	**436**	**282**
	28	Children	40			76	
	97	Adults	160			282	
	24	Seniors	48			78	
**Inpatient days**	**90**		**313**	**Days**	**733**	**230**	**148**
	17	Children	49			36	
	57	Adults	185			136	
	16	Seniors	79			58	
**Medication days**	**1024**		**13,512**	**Days**	**11–58**	**29**	**18**
	177	Children	1,966			5	
	747	Adults	9,754			21	
	100	Seniors	1,792			3	
**Minor procedures**	**301**		**314**	**Procedures**	**39–160**	**29**	**18**
	18	Children	20			2	
	261	Adults	270			24	
	22	Seniors	24			3	
**Radiology images**	**84**		**86**	**Images**	**179–385**	**17**	**11**
	8	Children	8			2	
	64	Adults	66			13	
	12	Seniors	12			2	
**Direct non-health care costs (transportations)**	**1151**		**2436**	**Round trips**	**18–133**	**240**	**155**
	202	Children	444			44	
	831	Adults	1722			169	
	118	Seniors	270			27	
**All costs**						**2,899**	**1,870**
	202	Children				421	272
	831	Adults				2,156	1,390
	118	Seniors				322	208

^*^All costs per one million population

The number of minor procedures was calculated for the three age groups as well as for all diagnostic groups. To calculate the costs of each case, we used the internal resourced-based HUEH cost accounting data (Table 2).

The unit cost of medication was obtained from the Finnish Medical Society’s national health portal [24].

The unit costs for transportation were obtained from the Social Insurance Institution of Finland, KELA, and the Department of Health and Welfare [25–27]. The total transportation cost was the sum of costs for two groups: patients receiving, and patients not receiving a travel allowance.

Lost productivity was estimated by multiplying the number of days absent from work by the employee’s average daily cost of lost productivity, for patients aged 17–64 and for one parent of the injured children needing physical restriction (Table 2). The number of days of lost productivity included the days of sick leave resulting from the eye injuries, imminent surgeries, admissions, and follow-up visits. The lost productivity costs were based on the wages of all employees in Finland added to the employers’ social security contributions and divided by the number of all employees [28, 29].

### Costs during follow-up

On the last visit, the data obtained included main abnormal status findings, outpatient admissions, days of hospitalization, major surgeries, minor procedures, medication, and computed tomography (CT) or magnetic resonance imaging (MRI). The unit and total costs by age group and the mean direct, indirect, and total costs during follow-up were estimated, in different diagnostic groups. The total cost per one million population was obtained. The costs were represented in euros, aligned with the Finnish health care producer price index 2020 [30].

### Estimating the future costs after follow-up

The estimations of the future costs after follow-up are presented in supplementary Tables 1–3, [36–40] and in supplementary Fig. 1 and discussed in the “Discussion” section.

This study was approved by the ethics committee of the Helsinki–Uusimaa Hospital District and followed the tenets of the Declaration of Helsinki.

## Results

### Resource use and costs during follow-up period

Of the 1151 patients, 202 were children aged under 17, 831 were adults aged 17–60, and 118 patients were over 60 years old.

#### Resource use

Table 2 shows resource use as follows:

- The number of outpatient visits was 2,436. Children needed 444 (mean 2.2), adults 1,722 (mean 4.1), and seniors 270 visits (mean 2.3).
- The number of major operations was 248 for 149 patients. Fourteen percent (28) of children, 12% (97) of adults, and 20% (24) of seniors underwent surgery. The number of minor procedures was 314 for 301 patients.
- Inpatient days were 313 for 90 patients. Eight percent (17) of children, 7% (57) of adults, and 14% (16) of seniors needed inpatient care.

Medication was used by 1,024 patients for 13,512 days (mean 13, range 1–215 days).

We estimated that 84 patients had to undergo 86 imaging of the head (76 CTs, 10 MRIs).

Based on the location of the patients living in HUEH area, 54.8–76% received a travel allowance of EUR 51.98/trip, after paying a deductible of EUR 9.25/trip. The transportation cost for patients not receiving the travel allowance was estimated to be EUR 9.25/trip [25–27].

A total of 6,902 days of lost productivity were needed for 825 people (mean 8.9; median 3; mode 3; range 1–313 days). From this amount of lost productivity, major operations caused 2470 days of lost productivity (100 operated patients of working age had 2,195 days, and 28 parents of operated children had 275 days).

#### Costs

**Fig. 1:**
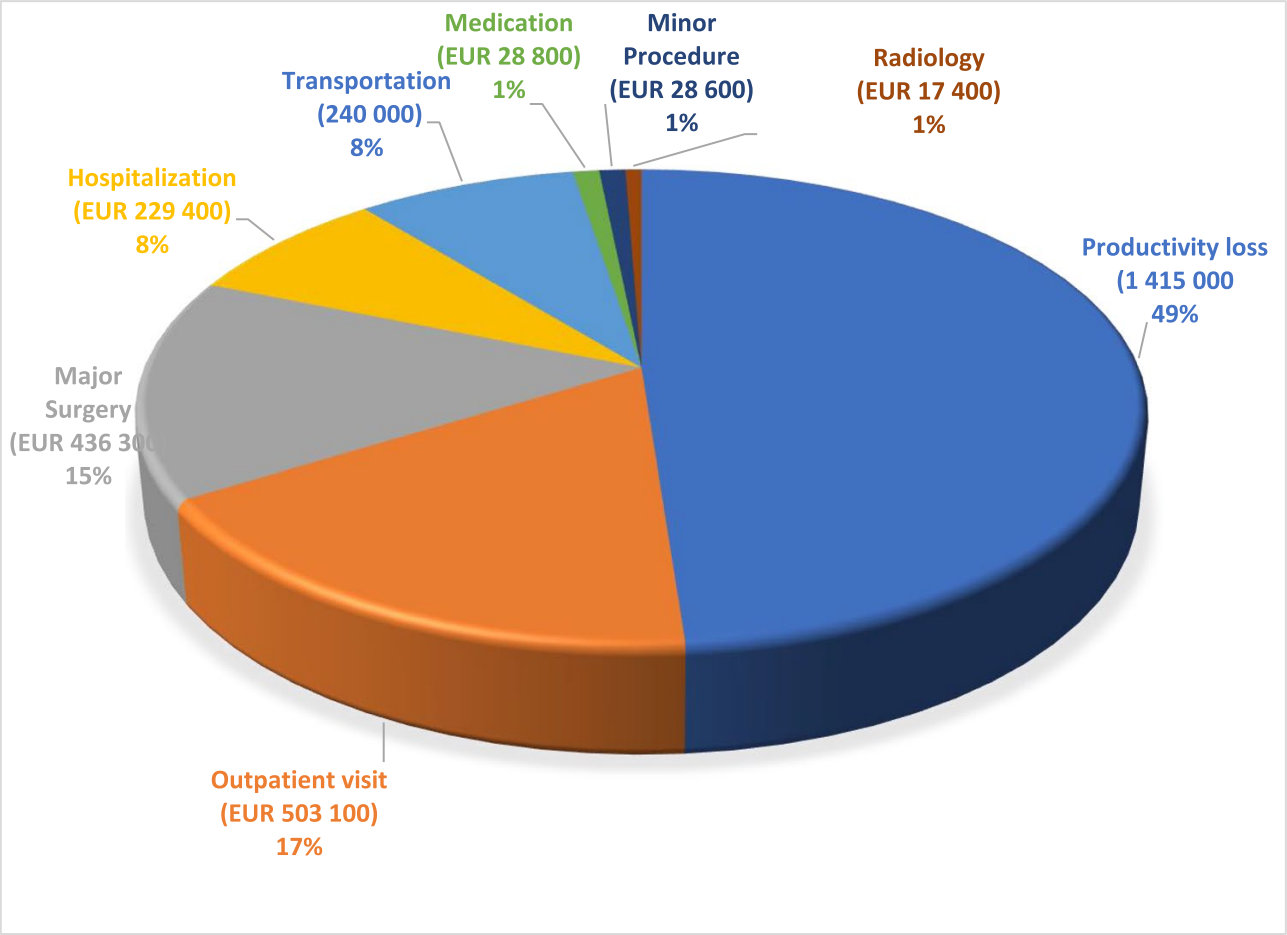
Cost of eye injuries during follow-up time in Southern Finland over a period of 1 year. Number of patients 1151, population base 1.6 million, follow-up 3 months

The total cost was EUR 2,899,000 for 1151 patients and EUR 1,870,000 per one million population. This included lost productivity (49%), direct health care (43%), and transportation costs (8%) (Fig. 1). The total mean cost was EUR 2,515/patient (Table 1).

Direct health care costs amounted to EUR 1,244,000 (Table 2). This included the costs of outpatient visits (41%), major surgeries (35%), inpatients (19%), medication (2%), minor procedures (2%), and radiology (1%).

The total transportation cost was EUR 240,000 (Table 2, Fig. 1).

The mean total cost was EUR 2,080/child, EUR 2,590/adult, and EUR 2,730/senior. The mean cost by diagnostic group varied between EUR 1,010 and EUR 13,420 per patient. The lowest cost was for minor superficial injuries at EUR 1,010/patient, and the two most costly diagnostic groups were OGI at EUR 13,420/patient and optic nerve injuries at EUR 10,490/patient (Table 1).

## Discussion

The present study shows that eye injuries cause a considerable economic burden. The costs of eye injuries were EUR 421,000 for children, EUR 2,156,000 for the adults, and EUR 322,000 for the elderly during the follow-up period of 1 year. A total cost of EUR 2,899,000 and 6,902 days (= 19 years) of lost productivity were incurred by all new eye injuries that occurred during the 1-year study period in a population of 1.55 million. Contusions (2,922 days, 42%) and superficial minor injuries (1,466 days, 21%) were the largest diagnostic groups that caused lost productivity.

An annual direct and indirect cost of USD 5 million (EUR 7,3 million [11]) and a loss of 60 work years was estimated among 3,184 patients with ocular injuries presenting to the Massachusetts Eye and Ear Emergency Service over a period of 6 months in 1985. This included outpatient visits and hospitalizations but excluded orbital and facial fractures [13].

In the present study, outpatient visits incurred the greatest cost among the direct health care costs, in the follow-up period.

The number of days as inpatient and as lost productivity caused by OGI has varied in previous studies. The mean length of hospital stay for perforating eye injuries in HUEH in Finland was 26 days in the 1950s [31], 20 days in the 1970s–1980s [8, 32], and 9.6 days in the present study. The mean length of lost productivity was 49 days in the 1970s [32], 90 days in the 1980s [8], and 54 days in the present study.

The health care cost level is lower in Finland than in the other Nordic countries and the USA [33, 34]. This may result in a relatively lower economic burden of eye injuries than in the mentioned countries. Comparison between reports on the costs of eye injuries is challenging, not only due to different health care cost levels, but also due to different study designs and focuses of interest.

A few other previous studies have reported data on the costs of hospitalization. According to Iftikhar et al., the median inpatient costs for eye injury in the USA was USD 11,000 (EUR 8000) in 2014 [14]. In Taiwan in 2001–2002, the mean hospitalization cost of a serious eye injury was USD 900–1,400 [15] (EUR 960–1,500) [11].

In the present study, during the follow-up period, the total mean costs per patient varied between EUR 1,010 for a minor superficial injury and EUR 13,420 for OGI. However, minor superficial eye injuries surprisingly had the second highest overall costs (EUR 609,600), after contusions (EUR 1.02 million), due to their highest occurrence among all diagnostic groups. In Croatia, the costs of minor eye injuries were EUR 135,500 in 2002–2003 [6].

We estimated the future costs incurred by the studied population (supplementary Tables 1–3 and supplementary Fig. 1).

The costs caused by 331/1,151 patients after the follow-up period (EUR 5,799,200) derived mainly from the high number of life-long follow-ups. After the follow-up, the estimated total mean cost per patient varied between EUR 470 for a minor superficial injury and EUR 20,110 for a contusion. The cost by a contusion was mainly due to the required high number of life-long follow-up visits (supplementary Tables 1–3). The total cost of eye injuries (EUR 8.7 million) during and after the follow-up, together, consisted of indirect costs for lost productivity (EUR 3.9 million, 44%), direct health care costs (EUR 3.4 million, 39%), and transportation costs (EUR 1.4 million, 17%) (supplementary Fig. 1). This total cost (EUR 8.7 million) corresponds to EUR 5.61 million/one million population (= costs during follow-up 1.870 million per one million population + estimated future costs 3.741 million per one million population).

The limitations of this study include, first, its short clinical follow-up time. A longer follow-up would give more accurate estimates of future required surgeries, admissions, and costs. The present study included only imminent future surgeries. Second, we lacked data on the costs of patients who are dependent on a caregiver (very old patients with a serious underlying disease, people with dementia, the disabled). Third, we lacked data on the far-reaching future economic burden caused by permanently impaired patients. As we reported previously [20–22], 107 patients (19 children, 73 adults, and 15 elderly people) had a permanent visual or functional impairment. The impact of this is apparent on one’s profession as reduced or lost productivity. Fourth, our estimations did not include costs of rehabilitation and vision aids such as eyeglasses and contact lenses. All these limitations underestimate the total costs of eye injuries. On the other hand, because of the use of the human capital method, the cost of productivity loss may be overestimated, and further studies are encouraged.

The future costs were presented in their nominal value; hence, discount rate was set to zero as the Finland Government Bond 10-year reference has been continuously negative starting from 24.4.2020 except a short period in May 2021 [35]. Discounting rate is ambiguous in the present state of the world economy. However, if previously used discount rates were used, the discounted present value of the future costs would be lower than in the present study.

One of the strengths of the present study is that it is a population-based study, as HUEH is practically the only ophthalmology acute care trauma unit in the Helsinki and Uusimaa Hospital District in urban and rural Southern Finland and covers approximately 29% of the population of Finland. Some minor eye injuries may have been treated in private care outpatient clinics, but these operate mainly by appointment and do not offer acute eye injury care or treatment. Another strength of the study is its size which was suitably representative of a sparsely populated country such as Finland. Other strengths are that the study reported the costs of both major and minor eye injuries in the clinical follow-up period as well as life-long costs.

In conclusion, eye injuries lead to considerable use of resources and costs in both the short and the long term. Knowledge of the burden and costs that eye injuries cause helps decision-makers set policies for priorities, helps prevent eye injuries, and may improve cost-efficiency.

Policies should be set to prevent eye injuries, based on the preventable nature of these injuries, at individual and community levels. Compliance with safety regulations and measures at work and at home should be increased by enhancing people’s general knowledge about the risks and costs of eye injuries. The detailed savings and health-promoting effects of prevention remain to be shown by further studies.

## Supplementary Information

Below is the link to the electronic supplementary material.

Supplementary file1 (DOCX 65.2 KB)

## Data Availability

The data that support the findings of this study are available on request from the corresponding author.
