# Supplementary material for: Resource use and economic burden of eye injuries in Southern Finland
Source: Graefes Arch Clin Exp Ophthalmol. 2021 Sep 6;260(2):637–43. doi: 10.1007/s00417-021-05399-3 (PMC8786766; doi:10.1007/s00417-021-05399-3)
Supplement: Supplementary file 1 — Supplementary file1 (DOCX 65.2 KB) [file 417_2021_5399_MOESM1_ESM.docx]

**Supplementary Tables and Figures**

**Supplementary Table 1.** The methods and assumptions for future cost estimations.

| **Cost component** | **Methods and assumptions** | |  |
| --- | --- | --- | --- |
| **Outpatient visits** | 2 sub-groups: life-long follow-up visits + surgery-related visits | |  |
| **Need for life-long follow-up visits** | All open globe injuries & contusions with intracameral hyphaema, or IOP>21, or detachment of retina^1^ | |  |
| **Number of life-long follow-up visits** | Based on assumption of annual life-long visit. Life-expectancy derived from Eurostat and Statistics of Finland; calculated from the median age of each age-group^2^ | |  |
| **Need for future surgery** | Included imminent & predictable cases.  E.g., cataract surgery based on post-traumatic posterior capsular opacification | |  |
| **Number of future surgery-related visits** | 3 visits per patient :  1 day for pre-operation + 1 day for post-operation + 1 day for the surgery | |  |
| **Number of days of medication** | Calculated only for patients with a need for future surgery | |  |
| **Number of transportations  (roundtrips)** | Assumed to be the same as the number of future outpatient visits | |  |
| **Number of days of lost productivity** | Days needed for life-long follow-ups + surgery-related sick leave days | |  |
| **Discount** | The future costs were presented in their nominal value | |  |
|  |  |  | |

^1^[References:32-34], ^2^[References:35-36].

**Supplementary Table 2.** Unit and total costs after follow-up of eye injuries by age group in Southern Finland.

| **Resource** | **Patients** | **Age Groups** | **Cost Component** | **Cost Component Units** | **Unit Costs^1^** | **Total Cost;** |
| --- | --- | --- | --- | --- | --- | --- |
|  | (n) |  | (n) |  | (€/unit) | **Thousands (€)** |
| **Indirect costs (Lost productivity)** | **305** |  | **11,887** | **days** | **205** | **2,436.8** |
|  | 64 | Children | 4,129 |  |  | 846.4 |
|  | 230 | Adults | 7,577 |  |  | 1,553.3 |
|  | 11 | Seniors | 181 |  |  | 37.1 |
| **Direct health  care costs** | **331** |  |  |  |  | **2,201.9** |
| **Outpatient visits** | **331** |  | **11,800** | **visits** | **150–250** | **2,124.0** |
|  | 64 | Children | 4,087 |  |  | 735.7 |
|  | 230 | Adults | 7,101 |  |  | 1,278.2 |
|  | 37 | Seniors | 612 |  |  | 110.1 |
| **Major operations** | **50** |  | **50** | **operations** | **710–2600** | **67.5** |
|  | 6 | Children | 6 |  |  | 8.1 |
|  | 34 | Adults | 34 |  |  | 47.4 |
|  | 10 | Seniors | 10 |  |  | 12.0 |
| **Inpatient days** | **10** |  | **10** | **days** | **733** | **7.2** |
|  | 2 | Children | 2 |  |  | 1.4 |
|  | 6 | Adults | 6 |  |  | 4.4 |
|  | 2 | Seniors | 2 |  |  | 1.4 |
| **Medication days** | **50** |  | **1560** | **days** | **11–58** | **2.15** |
|  | 6 | Children | 150 |  |  | 0.3 |
|  | 34 | Adults | 840 |  |  | 1.3 |
|  | 10 | Seniors | 570 |  |  | 0.55 |
| **Minor procedures** | **0** |  | **0** | **procedures** |  | **0** |
|  | 0 | Children | 0 |  |  |  |
|  | 0 | Adults | 0 |  |  |  |
|  | 0 | Seniors | 0 |  |  |  |
| **Radiology images** | **6** |  | **6** | **images** | **179–385** | **1.05** |
|  | 0 | Children | 0 |  |  | 0 |
|  | 6 | Adults | 6 |  |  | 1.05 |
|  | 0 | Seniors | 0 |  |  | 0 |
| **Direct non-health care costs (Transportations)** | **331** |  | **11,800** | **round trips** | **18-133** | **1,160.5** |
|  | 64 | Children | 4,087 |  |  | 401.9 |
|  | 230 | Adults | 7,101 |  |  | 698.4 |
|  | 37 | Seniors | 612 |  |  | 60.2 |
| **All costs** | 331 |  |  |  |  | **5,799.2^2^** |
|  | 64 | Children |  |  |  | 1,993.8 |
|  | 230 | Adults |  |  |  | 3,584.1 |
|  | 37 | Seniors |  |  |  | 221.3 |

^1^ Discount rate set to zero, ^2^ Corresponding to EUR 3,741,400 per 1 million population.

**Supplementary table 3**. Mean direct, indirect, and total cost after follow-up of eye injuries by different diagnostic groups. Follow-up 3 months.

|  | **Patients** | **Direct Health Care Cost** | **Direct Non-Health Care Cost** | **Patients** | **Lost Productivity** | **Indirect Cost** | **Mean**  **Total Cost** |
| --- | --- | --- | --- | --- | --- | --- | --- |
| **Diagnostic Group** | **(n)^1^** | **(€ / Pt)^2^** | **(€ / Pt)** | **(n)^9^** | **(days)** | **(€ /pt)** | **(€ /pt)** |
| **Chemicals^3^** | *18* | 2,450 | 1,235 | *18* | 254 | 2,890 | **6,575** |
| **Contusions** | *238* | 7,600 | 4,075 | *222* | 9,795 | 9,045 | **20,110** |
| **Fractures^5^** | *14* | 4,510 | 1,980 | *14* | 366 | 5,360 | **11,850** |
| **OGI^6^** | *29* | 7,970 | 3,940 | *22* | 1,125 | 10,480 | **19,860** |
| **ONI^7^** | *3* | 6,660 | 3,640 | *3* | 111 | 7,585 | **17,885** |
| **Superficials^8^** | *16* | 180 | 100 | *15* | 15 | 205 | **470** |
| **Wounds^9^** | *13* | 2,480 | 1,060 | *11* | 221 | 4,120 | **7,025** |
| ***ALL*** | ***331*** | **6,650** | 3,500 | ***305*** | **11,887** | **7,990** | **17,510** |

^1^N = number of patients; ^2^€/Pt = Euros per patient; ^3^Chemical & burn injuries; ^4^Orbital fractures; ^5^Open Globe Injuries; ^6^Optic Nerve Injuries; ^7^Superficial minor injuries; ^8^Eyelid wounds and/or canalicular injuries; ^9^number of patients with lost productivity.

**Supplementary figure 1.** Total costs of eye injuries during and after follow-up in Southern Finland over period of one year. Number of patients 1151, population base 1.6 million, follow-up 3 months.
